# Exploring the experiences of the team members in the interprofessional socialization process for becoming a interprofessional Collaborator

**DOI:** 10.1186/s12912-022-01147-y

**Published:** 2022-12-22

**Authors:** Fatemeh Keshmiri

**Affiliations:** 1 Medical Education Department, Educational Development Center, Shahid Sadoughi University of Medical Sciences, Yazd, Iran; 2 Faculty of Public Health, Shahid Sadoughi University of Medical Sciences, Yazd, Iran

**Keywords:** Interprofessional practice, Interprofessional collaboration, Interprofessional identity, Professionalism, Socialization, Interprofessional professionalism, Uni-professional

## Abstract

**Background:**

The current study aimed to explore the team members’ experiences in the socialization process for becoming a collaborator in an interprofessional team.

**Method:**

This qualitative study is conducted using an inductive qualitative content analysis approach. Participants consisted of 32 physicians (*n* = 16) and nurses (*n* = 16) who participated by purposeful sampling. Data were collected through in-depth semi-structured interviews and analyzed by Graneheim and Lundman approach.

**Results:**

In the study, “the perceived confrontation between interprofessional professionalism and uni-professionalism in the interprofessional socialization process” is explored as the theme, including two categories: “interprofessional professionalism commitment” as a facilitator and “uni-professional centrism” as a barrier.

**Conclusion:**

A reciprocal dimension in interprofessional socialization was explored. Interprofessional professionalism adherence and team-centered accountability among team members were explored as a facilitator. The uni-professional culture and immature interprofessional collaboration competencies of team members disrupted the interprofessional socialization process.

## Introduction

The ‘interprofessional collaborator’ is recognized as a critical role in working with different healthcare professionals in a team [1]. The role of ‘collaborator’ in health workers formed in the interprofessional socialization process. The workers of different professions in an interprofessional team experience socialization through working and learning from and about each other. *Socialization* is a process that begins in school and continues until the end of professional activity to form the professional identity of people [2]. Cruess et al. believed that identity is achieved when a worker demonstrates knowledge, competence, performance, and action as a professional [3]. Improving interprofessional knowledge, beliefs, and skills facilitates the formation of interprofessional identification [2].

Interprofessional socialization facilitates the development of a dual identity. The dual identity formation requires creating a sense of belonging to the profession and self-awareness as a member of the healthcare team/community [4]. Interprofessional socialization increases the preparedness of healthcare professionals for effectively integrating interprofessional collaboration into their current activities [4].

The interprofessional identity of healthcare workers helps to achieve the goal of interprofessional practice. Interprofessional practice occurs when healthcare workers from two or more professions work together with a common purpose, commitment, and mutual respect [5]. Reeves and colleagues explained the four forms of interprofessional practice including teamwork, collaboration, coordination, and networking. They believed The form of interprofessional teamwork required high levels of core elements consisting of shared team identity, clarity of goals and roles, interdependence, integration, and shared responsibility. Interprofessional teamwork facilitates the management of unpredictable, urgent, and complex situations such as emergency services. Interprofessional collaboration was a ‘looser’ form of interprofessional teamwork. Interprofessional collaboration required a high level of shared accountability and interdependence between individuals, as well, as clarity of roles/goals. The shared identity and integration of workers were less important in collaborative groups than in teams. Interprofessional coordination was similar to collaboration in terms of shared identity, shared accountability between workers, and clarity of roles and goals. However, integration and interdependence were viewed as less significant. In the format of interprofessional networks, shared team identity, clarity of roles/goals, interdependence, integration, and shared responsibility were viewed as less essential than coordination. The format of interprofessional networks matched with the situations where predictable, non-complex, and non-urgent care was required such as in a primary care practice setting. The clinical purpose and patients’ needs to direct the form of interprofessional practice according to the contingency approach [6].

Interprofessional socialization plays a key role in the interprofessional identification of workers and team success [7]. The interprofessional socialization process goes beyond the arrangement of different professionals [4] and is affected by individual, cultural, and contextual factors. According to the best of our knowledge, the explanation of the interprofessional socialization process using a qualitative approach is less addressed in the previous studies [4, 8–10]. McGuire’s study suggested further research on the reciprocal process of professional socialization and interprofessional socialization from the viewpoints of learners and personnel is needed [11]. It was suggested to use a qualitative approach to identify the factors affecting the interprofessional collaboration process [9]. This study aimed to explore the experience of healthcare team members related to facilitators and barriers in interprofessional socialization to become a collaborator.

## Methods

The present study was designed according to the qualitative content analysis introduced by Graneheim and Lundman [12]. The content analysis approach is suitable when new areas are to be investigated in an exploratory manner or if it has been decided to explore a known area from a fresh perspective [13]. The present study is a part of a large study that was conducted using grounded theory qualitative research to explain the interprofessional socialization process. Socialization is a complex phenomenon that needs to be investigated in an exploratory manner from a fresh perspective.

### Study setting and participants

The present study was conducted from June 2020 to September 2021 at teaching hospitals affiliated with Shahid Sadoughi University of Medical Sciences. The study was conducted in the Iranian context, where the participants experienced collaboration in the interprofessional team from the clerkship course in the educational period to the career era. The educational program used a uni-professional strategy in our universities. Inclusion criteria were the physicians and nurses who worked in the healthcare team in internal and emergency departments for at least 6 months, the experience of working in an interprofessional healthcare team, and willingness and readiness to participate in the study. Purposeful sampling was used in the study. To reach the maximum diversity of participants, they were entered from sexes, different professions (nursing and medicine), different job positions, and different experience levels. Participants consist of 32 physicians (*n* = 16) and nurses (*n* = 16).

### Data collection

Data were collected through in-depth semi-structured interviews. The interviews were conducted by a trained interviewer (Ph.D. graduate in medical education) with 5 years of experience in qualitative research. There was no defined relationship between the interviewer and participants. The interviewees’ choice of place and time was based on the participants’ suggestions. The duration of each session ranged from 35 to 65 minutes (a mean of 55 minutes).

The research aims were explained in the first session to obtain written informed consent. After obtaining permission and written consent, the interviews were recorded with a voice recorder. Each interview started with a warm-up and open-ended questions followed by probing questions. For instance, “would you please tell me how you participate in the process of interprofessional cooperation? “What factors encourage you to participate with them? What barriers affect one? Have you been able to develop a dual identity including professional identity and interprofessional identity? what helped you accept yourself as an interprofessional team member? what were the obstacles?”. After collecting data from 28 participants reached. Some participants were interviewed more than once. Six follow-up interviews were conducted to explain their experiences. In sum, 38 interviews were conducted. When data saturation was reached, no additional codes were found [14].

### Data analysis

Data were analyzed by the inductive content analysis introduced by Graneheim and Lundman [12]. Each interview was read several times, analyzed word by word, line-by-line, paragraph by paragraph, and labeled with initial codes. Memos were written in the process. The codes were classified according to similarities and differences in initial categories. The subcategories were classified as more abstract according to the characteristics and dimensions. Finally, a theme emerged through constant comparison.

### Rigor

In the present study was conducted several methods were used to ensure trustworthiness [15]. The credibility of the results was ensured by member-check, peer-check, expert-check, and prolonged, in-depth engagement with data. The participants were asked to review the explored results to ensure that the findings matched their experience. (Member-checking). In addition, peer-check was used to examine the extracted codes and categories from the data by the research team with experience in the qualitative content analysis approach (*n* = 2). As well as the experts in qualitative research (*n* = 2) audit the present results (external audit). Maximum variation in sampling regarding gender, age categories, and profession and experience of participants was deliberated. Memos were also identified to improve the accuracy of the findings. Constant comparisons were utilized to evaluate the semantic and structural coherence of the extracted results. A clear description of the context, the characteristics of the participants, the sampling process, data collection, and data analysis was presented to achieve the transferability criteria.

### Ethical considerations

The ethics committee approved the present study of the National Center for Strategic Research in Medical Education, Tehran Iran. (ID: IR.NASRME.REC.1400.094). The objectives and methods used in the study were explained to the participants. Participants were reassured that the interview content had been kept confidential and anonymous. They were assured that participation in the study was optional and that they could withdraw from the study.

## Results

**Fig. 1:**
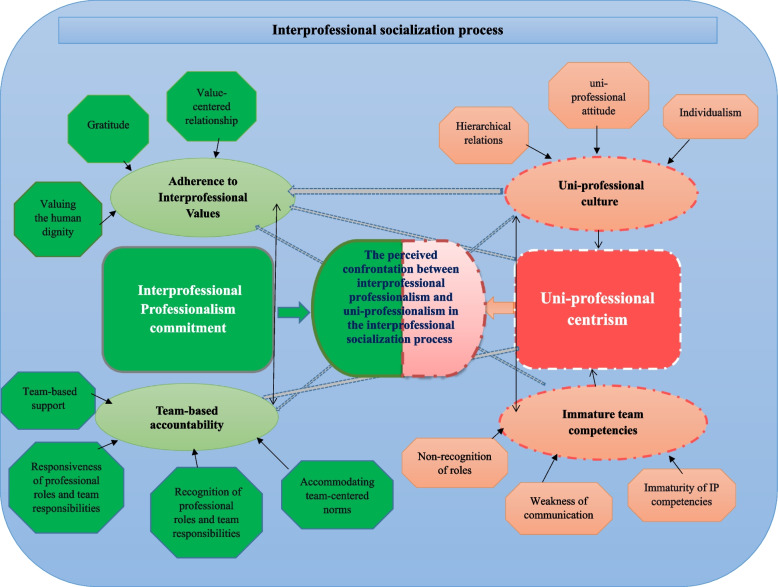
The explored conceptual framework of interprofessional socialization

Participants, including physicians and nurses (*n* = 32), contributed to the study. Their mean age was 38.5 ± 5, and most were female (*n* = 18). 1280 open codes were merged into four subcategories, two categories, and a theme. The influential factors of the interprofessional socialization process can be explained by ‘the perceived confrontation between interprofessional professionalism and uni-professionalism in the interprofessional socialization process.” “Interprofessional professionalism commitment” as facilitator and “uni-professional centrism” as a barrier were explored. (Fig. 1 and Table 1).

**Table 1:** The participants’ experiences regarding interprofessional socialization process

Subcategory	Category	Themes
Adherence to Interprofessional Values	Interprofessional Professionalism Commitment	**The perceived confrontation between interprofessional professionalism and uni-professionalism in the interprofessional socialization process**
Team-centered Accountability		
Immature Team-centered Competencies	Uni-professional centrism	
Uni-professional Culture		

### Interprofessional professionalism commitment

The adherence to ethical principles, professional commitment, and human and professional values ​​are perceived as facilitators in the process of interprofessional socialization. This category includes ‘adherence to interprofessional values’ and ‘team-centered accountability.’

#### Adherence to Interprofessional values

Creating value-centered relationships, gratitude, honoring the efforts of team members in interprofessional collaboration, and valuing the human dignity of personnel in the interprofessional socialization process were discussed in the subcategory.

Concerning respect for the team efforts of members in the interprofessional collaboration, a physician (a 32-year-old man) stated:

*“For me, being appreciative and reciprocal respect is more important than financial rewards; if people comprehend the merit of their work, they will cooperate well.”*

Concerning the role of respectful behavior with gratitude in developing interprofessional socialization, a participant stated:

*“In my idea, respect is the most important factor in interprofessional work. The work can be done much better by keeping respect for him/her. People need to realize the respect in interprofessional teams”. (A 33-year-old physician).*

#### Team-centered accountability

In this category, the golden factors for the effectiveness of interprofessional care were explored in the recognition, acceptance, and responsiveness of professional roles and team responsibilities—the subcategories deliberated responding to individual and team responsibilities, accommodating team-centered norms, and team-based support.

A participant related to the recognition and acceptance of individual and team roles stated:

*“If doctors recognize the role of the nurses as a moderator that facilitates the care process, they would better collaborate with nurses. Gradually, doctors and nurses develop a respectful and good relationship and cooperation.” (A 33-year-old physician).*

A participant regards responsibility and accountability to team duties stated:

*“I understand that I would do well when I could be with a team. I wanted to be a team member and do whatever I could. Now, I am more pleased with myself.” (A 35-year-old Physician).*

In this study, interprofessional norms meant the rules or principles that direct the behavior of team members in interprofessional collaboration. In this subcategory, acceptance, and respect for teamwork, such as commitment to interprofessional collaboration and learning, encouragement to teamwork, and development of perception toward team-based care, was considered the main factors in interprofessional socialization. Concerning team-centered beliefs, a participant stated:

*“Healthcare workers need to realize the clinical work is teamwork and must engage in the team. This perception solves teamwork issues. It matters.” (A 38-year-old Physician).*

Team-centered support means creating a positive and supportive environment among different professions. In this subcategory, the creation of multilateral support networks to develop interprofessional identity and achieve interprofessional collaboration was explored. These networks can be top-down hierarchies or cycles among members of a team. Managerial support, interprofessional education, and constructive feedback were explored as supportive factors.

Regarding the comprehensive support in the interprofessional collaboration, one of the participants stated:

*Helping and supporting teammates led to solving the problems and discriminations and creation of a supportive sense conveyed to the team for more effort”. (A 38-year-old nurse).*

### Uni-professional centrism

Two subcategories of ‘immature team-centered competencies’ and ‘uni-professional competence’ were explained as barriers to interprofessional socialization.

#### Immature team-centered competencies

This subcategory explored the barriers, such as immature interprofessional collaboration competencies in recognizing role and responsibility, interprofessional communication and teamwork, and issues about the uni-professional attitude.

Concerning immaturity of interprofessional competencies such as role recognition and interprofessional collaboration, a 34-year-old physician stated:

*“Specialists in Internal medicine do not collaborate with us because they do not accept us. They do not recognize and accept the role of emergency medicine at all.”*

A 34-year-old female nurse stated:

*“We work in the ward, but there is no collaboration. Everybody pursuing his/her goals; no common goal is pursued. We have neither learned nor implemented teamwork”.*

#### Uni-professional culture

Individualism is explored as an essential obstacle to becoming an interprofessional collaborator. The results showed that individualism and weak team-centered attitude inhibit acceptance and compliance with interprofessional principles and improve constructive teamwork among various professions. Some barriers to interprofessional socialization were a weak understanding of the interprofessional working nature, negative attitude, and team members’ preference for individualism instead of teamwork. A nurse said:

*“Physicians want to be brilliant themselves. In this competitive climate, they did not want to work in a team”. (A 36-year-old nurse).*

The promotion of a uni-professional culture and the factors disrupting the collaborative climate were explored as barriers to interprofessional identity formation in the socialization process. A 42-year-old male physician states:

*“Senior doctors told me: not to ask the nurses questions because they may think doctors depend on nurses. There was the perception that we do this, nurses will declare power, and then physicians will not be able to work with them”.*

The bordered interprofessional relations were explored as a challenging factor in the interprofessional socialization process. This category explores weak interprofessional communication skills and dictated hierarchical relations as barriers to interprofessional socialization. A 29-year-old female nurse said:

*“When we ask the doctor about something, they rush to say: ‘we will visit the patient by ourselves.’ You do not want to tell us what to do”.*

Another challenge emphasized is the ‘hierarchical relations in interprofessional teams.’ The impossibility of establishing direct communication among team members and the obligation to the communication hierarchy was explored as a barrier to teamwork.

*“I spoke with a doctor about a patient. He did not accept what I said. He referred me to the residents because he did not want to hear me as a nurse.* (A 28-year-old female nurse)*.*

## Discussion

The interprofessional socialization process prepares healthcare workers to play the role of interprofessional collaborators. The confrontation between interprofessional professionalism commitment and uni-professionalism is explored as reciprocal dimensions in interprofessional socialization. The formation of the interprofessional identity of workers is facilitated by their commitment to interprofessional professionalism and values and recognition and team-centered accountability. As well, uni-professional centrism through immature team-centered competencies and uni-professional culture disrupted the interprofessional identity process.

*Interprofessional identity* is defined as belonging to both professions and the interprofessional community [4]. The interprofessional identity is formed in interprofessional socialization as a process of building capacity in workers to form a dual professional identity and helping achieve interprofessional collaboration. Interprofessional socialization is defined as a process in which workers develop a dual professional and interprofessional identity (dual identity) through the acquisition of both professional and interprofessional beliefs, values, behaviors, and commitments to become an interprofessional collaborator. Interprofessional socialization facilitates the transformation of health professions education and practices toward effective interprofessional teamwork [16].

A study by Khalili and colleagues described a three-step process of interprofessional socialization. This process includes removing barriers, learning professional and interprofessional roles, and developing dual identities. Khalili’s model emphasized breaking down barriers related to the uni-professional perspective of workers to reduce/eliminate their out-group discrimination. The second stage of Khalili’s model focused on achieving the interprofessional competency domains to improve a sense of belonging and identity to the interprofessional team/community [16]. Consistently, the present results showed that the confrontation of uni-professional centrism and commitment to interprofessional professionalism as a main competency in the interprofessional socialization process was experienced by the healthcare workers. In Khalili’s model, the formation of interprofessional identity was shown as a linear model in three stages. In the present study, the formation of interprofessional identity was explained in the simultaneous confrontation between the commitment to interprofessional professionalism principles and uni-professional centrism. The predominance of each of the extracted factors plays an important role in the level of workers’ identification in the spectrum of interprofessional practice.

Reeves and colleagues defined the spectrum of interprofessional practice from teamwork to network. They showed that interprofessional practice is influenced by six elements including shared team identity, clear roles/goals, interdependence, integration, shared responsibility, and team tasks. The highest level of elements among team members facilitates the format of interprofessional teamwork and the lower level of those leads to the interprofessional network format [6]. Our results showed the increase in interprofessional professionalism commitment among workers had a curial role in the transformation of interprofessional practice into the interprofessional teamwork format. The predominance of uni-professional centrism resulted in forming an interprofessional network that did not match the needs of the emergency department.

Pettigrew and Troop in an intergroup contact theory discussed adherence to respect and value each other, and recognition of team working roles in a team effect on the practice of team members. In addition, they stated anxiety as feelings of threat and uncertainty that people experience in intergroup contexts required to decrease [17]. Similarly, our results indicated the components of interprofessional values ​​such as respect, value-based relationships, appreciation, and effective interprofessional relationships were explained, which can have a positive impact on the formation of interprofessional identity and also in achieving the goal of interprofessional practice. In our results, the uni-professional culture, issues about the uni-professional attitude, and individualism disrupted the interprofessional socialization process and increase the out-group anxiety. The uni-professional education in our context may result in increasing out-group conflict and negative stereotypes [18, 19].

Burford in a study discussed the group processes in medical education from the perspective of social identity theory. A main area in the social identity was defined as team-working as group processes in the workplace. According to the social identity theory, group membership was affected by positive attitudes towards in-group members and the denigration of out-group members. He stated healthcare workers often hold stereotyped views of one another and tensions can arise in different ways in a clinical setting [20]. Consistently, the present results explained the effect of conflict between the in-group and out-group in forming interprofessional identity. In line with our result, positive attitude and commitment to team norms were explored as facilitators, and individualism and uni-professional culture were explored as the main barriers in the interprofessional socialization of the healthcare workers.

Interprofessional professionalism underlined the adherence to the principles and values ​​such as respect, communication, excellence, altruism, trust and empathy, and responsibility of professional and team responsibilities within the interprofessional collaboration process [21, 22]. According to this result, compliance with professionalism principles and respect for professional values ​​such as mutual respect, gratitude, and accountability to team responsibility were explained as factors affecting interprofessional socialization. In the IPEC report, mutual respect constitutes the link in the interprofessional relationship for team-based care and plays a significant role in maintaining a respectful environment [1]. In line with our findings, Peu’s study showed that internalizing interprofessional shared values in the socialization process improves ethical, and collaborative performance in clinical environments [23]. The observance of respect and ethics, trust, integrity, and frankness in interprofessional collaboration explored the influential factors in their study [23]. According to our results, compliance with the professionalism principles among the team members facilitates the socialization for forming the interprofessional identity.

Team-based accountability was explored as the most important facilitator of interprofessional socialization in this study. This subcategory includes responding to individual and team responsibilities, accommodating team-centered norms, and team-based support. The recognition and acceptance of professional and interprofessional roles, responsibility, and accountability to individuals and teams were emphasized in this subcategory. The participants have stated that mutual understanding, the explanation of shared goals, teamwork, supporting each other, and efforts to respond to team needs played significant roles in forming interprofessional identities. The experiences of participants about the perceived facilitators were compatible with the competency domains reported by the Collaborative Interprofessional Education including ‘value and ethics’, ‘role and responsibilities, ‘team and teamwork, and ‘interprofessional communication [1]. In line with our study, an ethnographic study by Gaudet suggested that a sense of responsibility, communication in the collaborative process, and building mutual respect and trust were crucial elements of interprofessional collaboration [24]. Different studies acknowledged understanding the role and expertise of different professions were explored as a facilitator of interprofessional collaboration and respectful communication in the team [25–28].

The results showed adhering and respecting to team values highlighted in forming the interprofessional identity. The third step of the interprofessional socialization model that introduced by Khalili indicated people with dual identities value, respect, and celebrate a united team. Similar to the present study, adherence to the team-based norm was explored as a facilitating factor of interprofessional identity formation. A set of interrelated factors, including role recognition, team attitude, team support, and interprofessional commitment to collaboration, help the team members perform their professional duties and team responsibilities by following team norms. Consistent with the present study, Sims introduced team norms, shared and effective responsibility, and understanding of common goals as invisible factors affecting improved teamwork [29]. Soones and colleagues highlighted the teamwork culture as an influential factor in team-based care [30]. In line with the present findings, Schot’s results revealed that people of different professions collaborate differently. Their results indicated that interprofessional collaboration was established by eliminating professional social and physical problems, removing the barriers to professional duties via negotiation about overlapping roles and responsibilities, and creating opportunities to understand members’ professional duties and roles [31]. The present study explored horizontal and vertical support between the team and the organization as a facilitator of interprofessional identity formation. Team-based support means planning, establishing, and supporting a collaborative environment to meet the shared organizational goals and direct team members and the organization to work together to achieve the goals [24, 32]. Thus, the creation of interprofessional norms and the development of critical competencies among members of healthcare teams could significantly contribute to interprofessional identification.

In the next category, the factors disrupting interprofessional socialization were explored. Immature team-based competencies and uni-professional perceptions were two main factors disrupting the formation of interprofessional identity. The uni-professional centrism resulted in the acquisition of norms, values, beliefs, and the professional-centrism culture, without attention to the interprofessional culture. The dominancy of a uni-professional identity was explored as an obstacle to the interprofessional socialization process. Similarly, the social identity and intergroup contact theories discussed an isolationist approach that led to the development uni-professional identity of learners comprised of ‘in-profession favoritism’ and ‘out-profession discrimination’. These in-group and out-group behaviors may achieve due to limited understanding and knowledge of different professional roles. Their limitation is restricted to the participation of workers in interprofessional collaborative practice [16, 17]. In our context, uni-professional education was used in clinical education in formal and informal programs, and continuous education may result in the dominancy of the uni-professional identity among the workers. Similarly, profession-centrism was described as a barrier to social identity in interprofessional practice [33].

The present results showed the uni-professional culture, which arises from bounded relations and a negative attitude toward interprofessional collaboration among team members, disrupted interprofessional socialization. Individualism of team members, failure to understand the nature of interprofessional work, a biased attitude towards other professions, and stereotypes break down the interprofessional identification process. The ineffective communication [34] and conflicts among disciplines [35] hinder multilateral communication and team interactions that prevent effective collaboration [36]. In addition, stereotypes and prejudices among members of different professions reduced collaboration [37, 38]. These participants believed that the discriminatory atmosphere in the clinical wards due to the uni-professional culture obstructed the establishment of interprofessional identification. The effect of this atmosphere on other members, especially the new team members, led to the failing development of interprofessional identification. Similarly, Strudwick et al. believed that creating tribal and guild boundaries among different professions keeps members of different professions away from each other in the team and disrupt the interprofessional collaboration atmosphere [39].

Poor communication and collaboration with other healthcare team members were discussed in the category of immature Team-centered competencies. Perceived hierarchy and unawareness about the role and responsibilities of team members were introduced as the challenges of interprofessional cooperation in several studies [39, 40], which resulted from uni-professional centrism. As for the present study, Paradis’ study showed that failure to understand the professional hierarchy, roles, expertise, and performance could negatively affect service providers in the ward [41]. Our study showed weak attitudes and skills in interprofessional collaboration were the main barriers to interprofessional socialization. Lack of communication and teamwork skills, unawareness of the role of other people, discriminatory attitudes, and stereotypes create an atmosphere not conducive to establishing interprofessional identity. Pecukonis discussed the elimination of profession-centrism as a solution to achieve interprofessional practice among healthcare workers [33].

In the theoretical lens of signature pedagogy, three apprenticeships of learning have described the formation of identity; “ [1] a cognitive apprenticeship to learn to think like others in your profession, [2] a practical apprenticeship to learn how to perform like those in your profession and, [3] a moral apprenticeship to learn how to act with moral integrity” [42]. The cognitive and practical apprenticeship in the uni-professional setting reinforced the profession-centrism and disrupted the interprofessional identity formation among workers in our study. These issues may affect the resistance of workers to assume an interprofessional collaborator role in these teams. The participants acknowledged the uni-professional attitude, and individualism resulting from a cognitive apprenticeship in the uni-professional setting, that they learned to think uni-professionally. Moreover, the interprofessional competencies were learned by the practical apprenticeship that the workers learned from their professions in the uni-professional education and practice. The results of Best and colleagues showed the cognitive apprenticeship explored as the main challenge in the formation of professional identity. They believed that thinking as a member of an interprofessional team was a challenging issue while acting with moral integrity was more forthright [43]. Shulman (2005) highlighted professional education assists in the formation of behaviors of workers in the future, and facilitate understanding of values and constructs within their professions and interprofessional relationship [42]. Hence, the workers need support for preserving their professional identity and developing interprofessional identity through the fluidity of interprofessional working in a healthcare team. The interprofessional learning situations facilitated the exploration of the various facets of professional identity by the workers [43]. Interprofessional education was suggested to improve the team working in medical education systems by increasing understanding of other professions and reducing negative stereotypes [20].

We have addressed the experiences of healthcare workers related to the interprofessional practice of in-group and out-group. Further studies need to focus on the workers’ perception related to patients as a member of the team in interprofessional practice.

### Limitations

Because a qualitative approach was used in this study, the qualitative findings may not apply to other populations with different cultural backgrounds.

## Conclusion

This study discussed the factors affecting interprofessional socialization in becoming a collaborator. The perceived confrontation between interprofessional professionalism commitment and uni-professional centrism is explored as reciprocal dimensions in interprofessional socialization. Adherence to values ​​and professionalism play an essential role in interprofessional socialization among team members. The results indicated that interprofessional professionalism elements such as value-based relationships and communication, gratitude, respect, and team-based accountability facilitated interprofessional socialization. Immature interprofessional collaboration competencies and uni-professional culture among team members lead to the breaking down of the formation of interprofessional identification.

## Data Availability

The datasets generated and/or analyzed during the current study are not publicly available due to the confidentiality of the data of participants but are available from the corresponding author at reasonable request.
